# Robot-assisted pancreatic surgery—optimized operating procedures: set-up, port placement, surgical steps

**DOI:** 10.1007/s11701-021-01297-2

**Published:** 2021-09-02

**Authors:** Matthäus Felsenstein, Karl H. Hillebrandt, Lea Timmermann, Mathilde Feist, Christian Benzing, Moritz Schmelzle, Johann Pratschke, Thomas Malinka

**Affiliations:** 1 Department of Surgery, Charité-Universitätsmedizin Berlin, Corporate Member of Freie Universität Berlin, Humboldt-Universität zu Berlin, and Berlin Institute of Health, Campus Virchow Klinikum I Campus Charité Mitte, Augustenburger Platz 1, 13353 Berlin, Germany; 2 Clinician Scientist Program, Berlin Institute of Health (BIH), Anna-Louisa-Karsch-Str. 2, 10178 Berlin, Germany

**Keywords:** Robotic-assisted pancreatic surgery, Pancreatic surgery, Standard operating procedures, One fits all, Reversed 6-to-6 approach

## Abstract

Even in most complex surgical settings, recent advances in minimal-invasive technologies have made the application of robotic-assisted devices more viable. Due to ever increasing experience and expertise, many large international centers now offer robotic-assisted pancreatic surgery as a preferred alternative. In general however, pancreatic operations are still associated with high morbidity and mortality, while robotic-assisted techniques still require significant learning curves. As a prospective post-marketing trial, we have established optimized operating procedures at our clinic. This manuscript intends to publicize our standardized methodology, including pre-operative preparation, surgical set-up as well as the surgeons’ step-by-step actions when using pancreatic-assisted robotic surgery. This manuscript is based on our institutional experience as a high-volume pancreas operating center. We introduce novel concepts that should standardize, facilitate and economize the surgical steps in all types of robotic-assisted pancreatic surgery. The “One Fits All” principle enables single port placement irrespective of the pancreatic procedure, while the “Reversed 6-to-6 Approach” offers an optimized manual for pancreatic surgeons using the robotic console. Novel and standardized surgical concepts could guide new centers to establish a robust, efficient and safe robotic-assisted pancreatic surgery program.

## Background

Even in most challenging surgical interventions of the retroperitoneum, minimally invasive techniques are increasingly evaluated for their feasibility and efficacy. Indications for laparoscopic interventions have already demonstrated advantages over open pancreatic surgeries in some instances [1, 2]. Results of robotic-assisted interventions from high-volume centers suggest even broader application [3–5]. Due to high costs, the application of these technologies is limited to a few large international centers, so that universally applied Standard Operating Procedures have not yet been established. Some studies also indicate that significant center-specific and time-dependent differences prevail, thus any benefits of robotic-assisted pancreatic surgery may develop only after extended learning curves [6–9]. However, company marketing and the general popularity of such technologies increase daily. To minimize the rate of any serious complications, which may occur during the learning curve, standardization in experienced centers is extremely important. From our high-volume center-specific experiences, we present the following Optimized Operating Procedures, for the setting of robotic-assisted pancreatic surgery. This protocol should enable other centers to establish a robust-, time efficient- and safe robotic-assisted pancreatic surgery program.

## Logistics and informed consent

Robot-assisted technology is novel, so that it is necessary to explain to the patient both the risks and advantages compared to conventional operating methods. We generally inform patients about current international opinions and studies to provide individual, evidence-based recommendations when considering this surgical technology's pros and cons.

This prospective post-marketing study (CARE study; E/A4/084/17) was conducted with IRB approval, which was necessary to collect data of a unique cohort receiving surgical treatment with the da Vinci Xi (Sunnyvale, USA) Surgical System (DRKS00017229).

## Hospitalization

Initial surgical assessment and indication for surgery is obtained at our Charité Pancreatic Outpatient Center. Specialized and experienced pancreatic surgeons inform and consent selected patients for robotic-assisted surgery. Anesthesiologists are consulted to thoroughly examine the patient before scheduling the operation. In the context of a professionalized ERAS (enhanced recovery after surgery) program, patients are surveilled before and after surgery by expertly skilled personnel. Patient admission to the hospital occurs one day prior to the operation for final clinical evaluation.

## The positioning of the patient

**Fig. 1:**
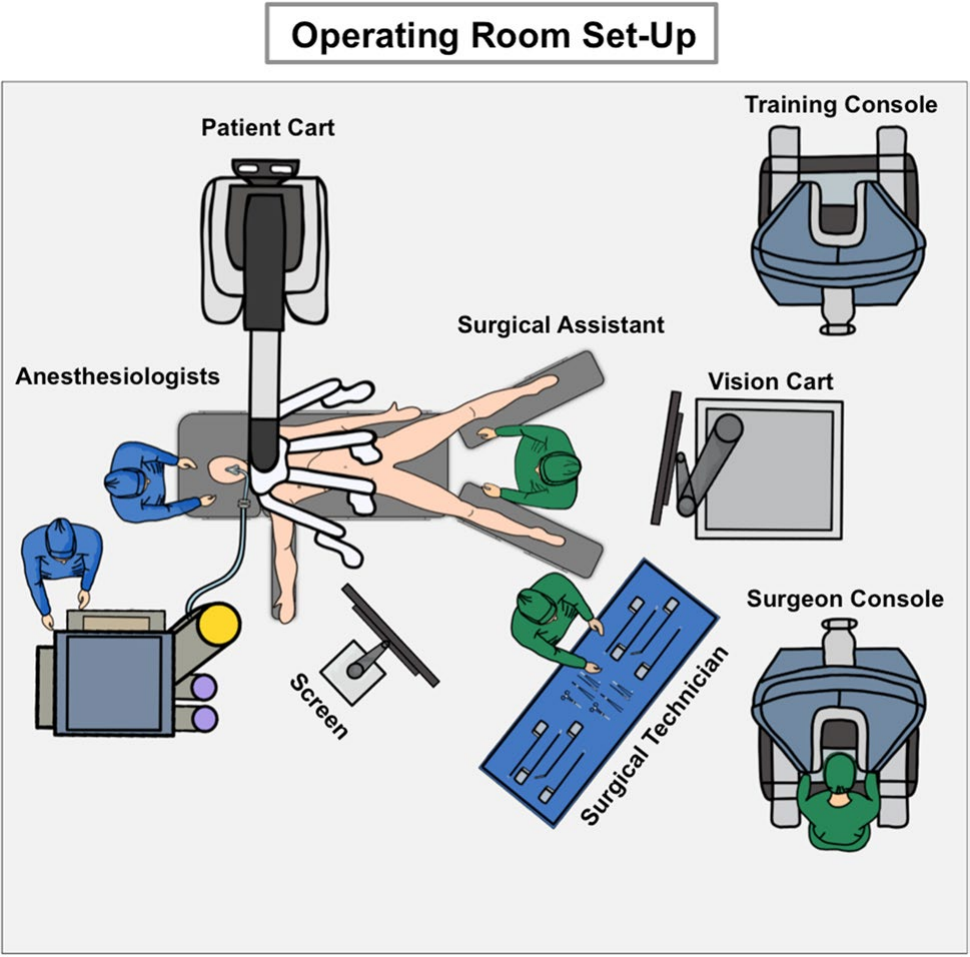
The operating room set-up: The set-up of the operating room is essential for process optimization and facilitated communication during the robotic-assisted procedure. We were able to limit the number of team members to adjust to any spatial constraints when working with robotic devices. This can be easily re-organized to address center-specific demands

To avoid positioning and collision injuries, patients are placed on a soft vacuum mattress, and metal protectors are attached for safety and shield the surgical area. The left arm is positioned at the patient's side, while the right arm is stretched out to give anesthesiologists easy access (Fig. 1). The patient's legs are placed in French Position while their body remains in 12° reverse Trendelenburg inclination.

## The positioning of the ports

**Fig. 2:**
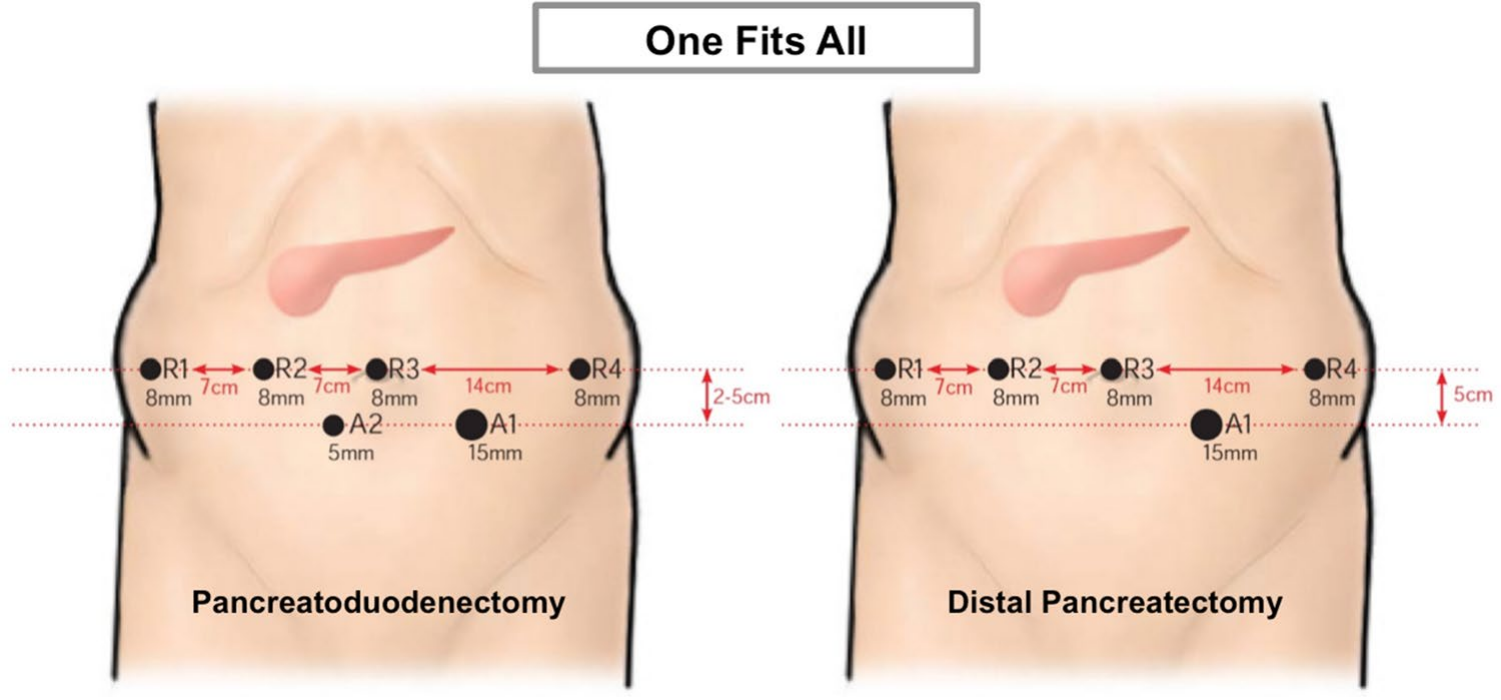
Port placement: Following the principle “One Fits All”, all trocars are positioned the same way irrespective of the pancreatic procedure. We start with the umbilical R3 position for diagnostic laparoscopy. We sequentially place trocars R1, R2 and R4 along a horizontal trajectory. Distances R1–R3 measure 7 cm to one another, while R4 is laid out at the left hemi-abdominal side in double distance (14 cm). Ultimately, assistant trocars (A1, A2) are positioned 3–5 cm below the umbilical horizontal line. The set-up for DP only differs in the lack of a second assistant trocar (A2)

Irrespective of the pancreatic procedure, we set out all the robotic and assistant trocars prior to attachment of the robotic arms. Our center has established a "One Fits All” principle. When performing pancreatoduodenectomies (PD), total pancreatectomies (TP) and Appleby procedures (AP), four robotic trocars (8 mm) and two assistant trocars (15 mm/5 mm) are needed. For Distal Pancreatectomies (DP) however, we introduce a single assistant trocar (15 mm). Figure 2 indicates the exact port placement.

First, we place 8 mm trocar (R3) umbilical. After a pneumoperitoneum has been established, a diagnostic laparoscopy is then performed. If there is a good overview, other robotic trocars (R1, R2, R4) are placed in an imaginary horizontal line using standardized distances to avoid robotic arm collisions (see Fig. 2). During this process, any intra-abdominal adhesions are removed using laparoscopic instruments, before introducing the remaining assistant trocars (A1, A2).

## Alignment of the da Vinci patient cart

**Fig. 3:**
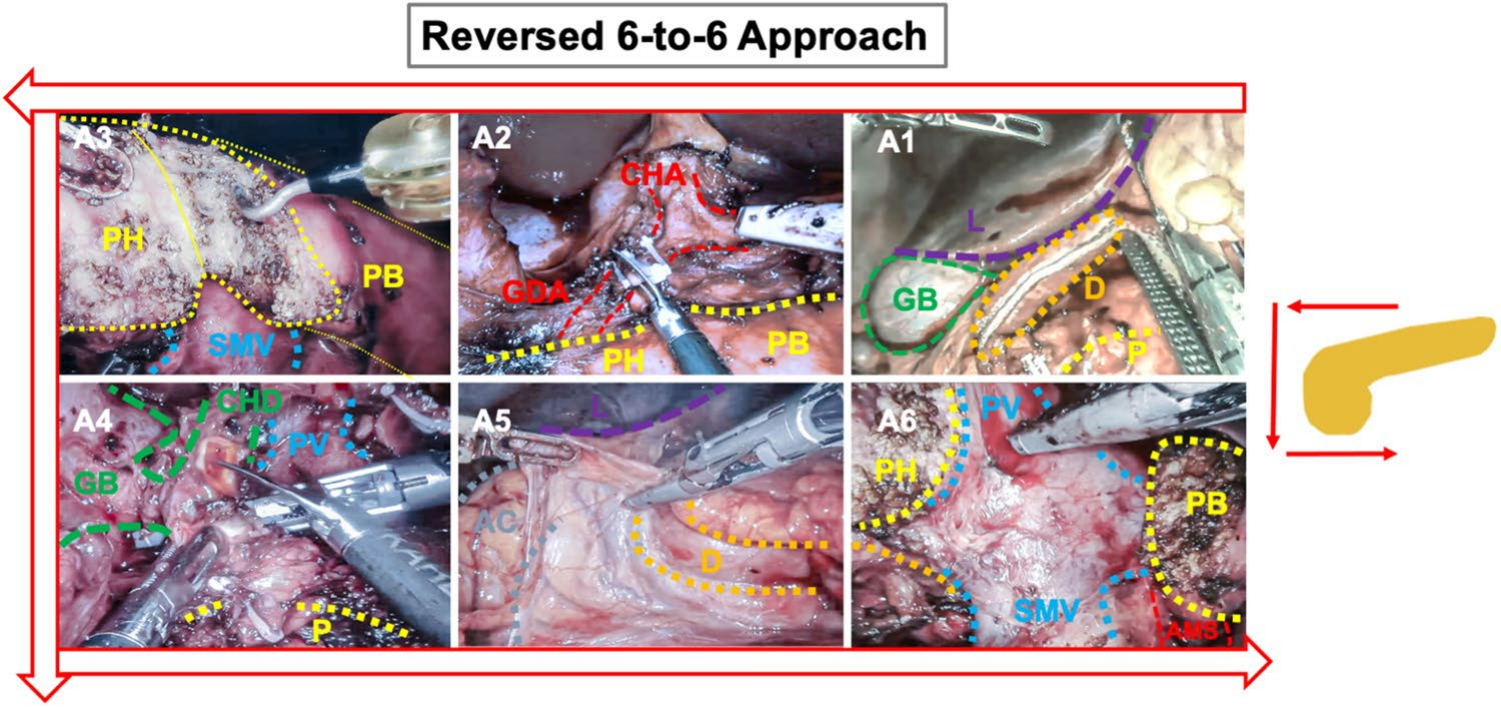
The “Reversed 6-to-6 Approach”: following the “Reversed 6-to-6 Approach”, we optimized the surgical steps best suited for robotic-assisted pancreatoduodenectomy. We start dissecting directly at the pancreas, before releasing the specimen from surrounding structures in anti-clockwise orientation. This systematic approach allowed us to economize operating time. *L* liver, *D* duodenum, *GB* gallbladder, *CHA* common hepatic artery, *GDA* gastroduodenal artery, *P* pancreas; *PH* pancreatic head, *PB* pancreatic body, *PV* portal vein, *CHD* common hepatic duct, *AC* ascending colon, *SMA* superior mesenteric artery

The da Vinci Xi (Sunnyvale, USA) Patient Cart is aligned with the operating table on the left side (Fig. 1). The camera is now being introduced (R3). After the focal point has been adjusted intra-abdominally, the robotic system and arms are set up fully automatic. On demand, the required instruments are introduced into the patient and connected with the robotic arms (Fig. 3) (Table 1).

**Table 1:** Robotic ports and instruments: robotic arms are connected with specialized instruments for robotic-assisted surgery

Trocar	Port	Size (mm)	Instrument
Robotic trocar	R1	8	Tip-up Fenestrated Grasper
Robotic trocar	R2	8	Fenestrated Bipolar Forceps
Robotic trocar	R3	8	Endoscope
Robotic trocar	R4	8	Vessel Sealer Extend, Permanent Cautery Hook
Assistant trocar	A1	15	Forceps, Scissor, Covidien EndoGia
Assistant trocar	A2	5	Forceps

Most instruments are used in defined port positions but may be adjusted and customized during each phase of the operation. According to the "One Fits All" principle, we list the distinct trocar/port positions coupled with common robotic instruments used in that position

## Steps for structured robotic-assisted pancreatoduodenectomy (PD)

Operating procedures have now been significantly optimized and economized based on our center-specific experience. During the period of 2017–2021, we were able to conduct > 125 robotic-assisted pancreatic surgeries and > 70 PDs. We believe that this process optimization is particularly important in the robotic setting due to its limited spatial overview when compared to the open situs operations. For this reason, our team established a novel concept, named the “Reversed 6-to-6 Approach”:

### A: Resection

#### A1: Entering the bursa omentalis to expose the pancreatic organ

When dissecting the greater omentum at the gastrocolic ligament, which enables the access to the bursa omentalis, the Tip-Up Fenestrated Grasper (R1) is utilized. The stomach is mobilized using the Fenestrated Bipolar Forceps (R2) as well as the Vessel Sealer Extend (R4) for dissection of the ligament. The stomach is then dissected directly at the post-pyloric plane using a Covidien (Dublin, Ireland) EndoGia (A1) with a purple cartridge (60 mm). The result is that the stomach can be mobilized into the upper-left quadrant providing an optimal view early on.

#### A2: Exposing the pancreas at the resection level

The next step includes dissection of the pancreas at the caudal edge to expose the mesenterico-portal axis. While retracting the liver using the robotic arm 1, Fenestrated Bipolar Forceps (R2) and the Vessel Sealer Extend (R4) instruments are needed for careful dissection. Additional support is provided using instruments via assistant trocars (A1, A2). Subsequently, cranial structures are exposed by conducting complete lymphadenectomy with the dissection of all-important vessel structures within the hepatoduodenal ligament (Common hepatic artery, CHA/Gastroduodenal artery, GDA/Right gastric artery, RGA). Identification of the GDA branching-point enables a safe ligation of the artery via Hem-o-lock clips (A1). The portal vein structure is then fully exposed at the cranial edge. At this point, the pancreatic body can be mobilized along the mesenterico-portal axis.

#### A3: Cutting the pancreas at the resection level

Following the mobilization from cranial and caudal edges, the pancreas is then carefully dissected along the mesenterico-portal axis using the Permanent Cautery Hook (R4).

#### A4: Preparation of hilar structures

Along the “Reversed 6-to-6 Approach”, the hilar structures can subsequently be dissected and visualized. Identification of the hepatocholedochus duct enables dissection right behind the main cystic branch using a scissor (A1). Subsequently, we perform anterograde cholecystectomy using the Permanent Cautery Hook (R4).

#### A5: Kocher Maneuver

The Kocher Maneuver is realized using the Fenestrated Bipolar Forceps (R1) and Vessel Sealer Extend (R4) instrument. These are progressed along the duodenum in a cranio-caudal direction until reaching the ligament of Treitz. The release of these latter segments allows the flection of the jejunal loop into the right upper quadrant. The jejunal loop is subsequently parted using the Covidien (Dublin, Ireland) EndoGia with purple cartridge (45 mm) (A1) to establish the alimentary loop.

#### A6: Completion at the mesenterico-portal axis

Finally, the dissection of the pancreatic head and the uncinate process is completed along the portal vein and superior mesenteric artery (AMS) using the Fenestrated Bipolar Forceps (R1) and Vessel Sealer Extend (R4). Small branches are clipped (A1), and the resection specimen is removed using a retrieval bag.

### B: Reconstruction

#### B1: Reconstruction of the hepaticojejunostomy

The alimentary loop is commonly opened at counter mesenteric position using the Permanent Cautery Hook (R4), followed by anastomosis of the hepaticojejunostomy in back-to-front direction using a continuous PDS suture (5–0), realized by Large Needle Driver (R4) und Tip-Up Fenestrated Grasper (R2). Prior to closure of the hepaticojejunostomy, a trans-anastomotic stent (2mm × 3cm) is positioned (PDS 5/0) to ensure biliary drainage.

#### B2: Reconstruction of the pancreato-gastrostomy

A suitable position for anastomosis is marked at the back wall of the stomach using the Permanent Cautery Hook (R4). A Prolene 5/0 suture along the incision line in purse-string technique is applied using the Large Needle Driver (R4) and Tip-Up Fenestrated Grasper (R2). The stomach is now incised using the Permanent Cautery Hook (R4), and when a clear view of the back wall is achieved, a robotic-adjusted pancreatogastrostomy is executed using our recently developed mattress-seam technique (Vicryl 3/0) [10]. The procedure is conducted with the Tip-Up Fenestrated Grasper (R2), and Large Needle Driver (R4) and a trans-anastomotic splint (2mm × 3cm) is positioned into the pancreatic duct. By pulling the mattress-seam sutures, the pancreatic tail is directly drawn into the stomach, fully covered by gastric mucosa. Finally, the outer pancreatogastrostomy is tightly sealed by purse-string sutures.

#### B3: Reconstruction of the gastroenteric anastomosis

Antecolic gastroenterostomy finalizes the reconstruction using continuous V-Loc 4-0 sutures. Again, the surgeon proceeds in a back-to-front direction using the Tip-Up Fenestrated Grasper (R2) and Large Needle Driver (R4) instruments.

#### B4: Disconnection of da Vinci robotic system

Before finalizing the procedure, the abdominal situs is inspected for minor bleeding, cauterized and rinsed to remove any remaining intraabdominal debris (R2). The instruments are removed under careful observation, and all robotic arms are disconnected before the entire patient cart is pulled back from the patients’ site. Subsequently, the bag containing the resection specimen is removed through a 5 cm mid-line retrieval incision. We also use this incision to conduct a haptic re-evaluation of all anastomoses, or, in rare circumstances, even to perform the reconstruction of the gastroenteric anastomosis entirely via this incision (hybrid approach). This allows for more flexibility during the initial stages of the learning curve.

#### B6: Drains and closure

As our institutional standard, we place drains through trocar incisions, which scan the regions around the pancreatogastrostomy and the hepaticojejunostomy (R4 position). The integral planes of mid-line retrieval incision and trocar wounds are closed with sutures.

## Steps for structured distal pancreatectomies (DP)

The new standard for non-oncologic distal pancreatectomies is the minimally invasive resection of the pancreatic tail [11]. With this in view, we sought to establish standard, robotic-assisted distal pancreatectomies at our center. With respect to the individual surgical indication (benign versus malign), this procedure can be conducted with spleen preservation (Kimura Maneovre) or via complete oncologic clear-up [12]. As spleen-preserving procedures are rarely performed, we describe the individual steps of an oncologic distal pancreatectomy.

### A: Resection

#### A1: Entering the bursa omentalis to expose the pancreatic organ

When dissecting the greater omentum at the gastrocolic ligament, which enables good access to the bursa omentalis, we use the Tip-Up Fenestrated Grasper (R1) instrument to mobilize the stomach and the Vessel Sealer Extend (R4) for dissection of the ligament. This is performed progressing from the right-medial peri-gastric plane to the left colic flexure using the Fenestrated Bipolar Forceps (R2) und Vessel Sealer Extend (R4) instruments, while the short gastric arteries are regularly dissected. Instruments maneuvering through assistant trocars (A1, A2) may provide additional support and a better overview. After full mobilization of the stomach, we recommend fixing the stomach via a single-armed suture with a straight needle, which we introduce sub-xiphoidal. It is then transfixed at the greater curvature and tied from the outside after the needle is re-released sub-xiphoidally. This enables an excellent overview of the bursa omentalis and the entire pancreatic organ.

#### A2: Exposing the splenic vessels

For malignant indications, an exact localization and planning of the resection plane is necessary and can be realized intra-abdominally via an ultrasound device (A2). At the resection level, the splenic artery is exposed at the cranial pancreatic edge, using the Fenestrated Bipolar Forceps (R2) and Vessel Sealer Extend (R4) instruments. Next, Hem-o-lock clips are introduced for safe ligation of the artery (A1). The splenic vein is commonly found at the pancreatic body's caudal edge, which is equally ligated via Hem-o-lock clips (A1). The pancreas can now be inflected from the Gerota fascia beneath.

#### A3: Cutting the pancreas at the resection level

Subsequently, the Covidien (Dublin, Ireland) EndoGia (A1) with a black cartridge (60 mm), reinforced by Seamguard Mesh, is introduced and tunneled at the resection site and dissects the pancreas at the required position.

#### A4: Retrieval of the resection specimen

The specimen can be released after thorough preparation of the pancreatic tail. It includes lymphadenectomy and mobilization of the spleen, using the Fenestrated Bipolar Forceps (R2) and Vessel Sealer Extend (R4) instruments. The specimen is transferred into a retrieval bag and recovered using an extended incision at position A1.

#### B6: Drains and closure

As an institutional standard, we place drains through trocar incisions, which scan the regions around the resection margin and the Koller's Pouch (R4 position). Integral planes of retrieval incision and trocar wounds are sutured to close them.

## Outcome parameters using optimized operating procedures

**Fig. 4:**
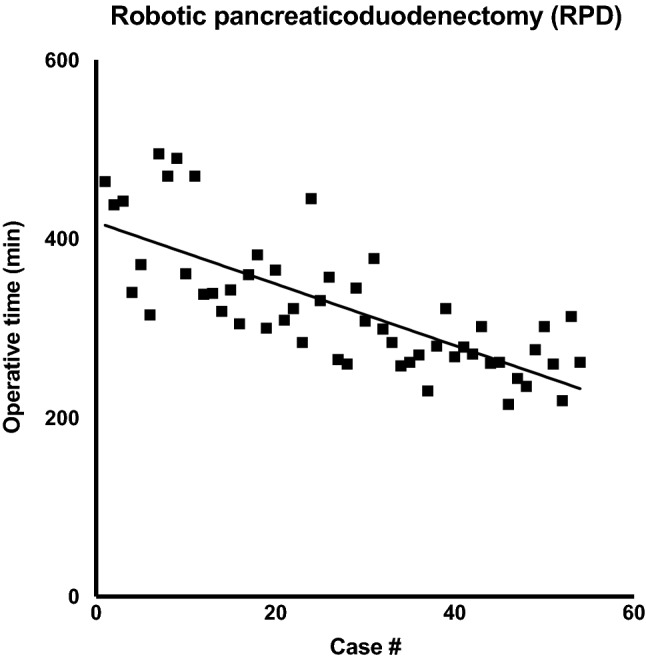
Institutional learning curve: regression analysis of operating time over the course of 54 RPDs as a result of process optimization. Overall, we were able to halve procedure time over the course of our first series, reflecting a particularly steep learning curve

In the recent series of robotic-assisted pancreatectomies at our center, we were able to report good outcome parameters, after having implemented novel concepts, such as the “One Fits All” principle and “Reversed 6-to-6 Approach” [4]. Of particular relevance in the evaluation of this novel approach are the parameters, such as mean procedure time, in-hospital stay, complication rates and oncologic outcomes (Table 2). Like other large international centers, we present similar positive outcome parameters, while offering a standardized approach, feasible for majority of patients. There is also a measurable reduction in operating time compared to other centers, likely due to our steep learning curve (Fig. 4). Altogether, this indicates successful process optimization through standardization.

**Table 2:** Clinical outcome parameters: main outcome parameters after robotic pancreaticoduodenectomy from various international centers were derived from published reports

	Charité Universitätsmedizin Berlin, Timmermann et al. 2021 [10]	University of Pittsburgh Medical Center, Zureikat et al. 2020 [9]	University of Hongkong, Zhang et al. 2019 [7]	Johns Hopkins University, Van Oosten et al. 2020 [8]	Shanghai Jiaotong University SOM, Shi et al. 2020 [6]
Case number	54	500	100	96	200
Procedure time (min)	325	415	358	474	279
In-hospital stay (days) 15	8	18	8	21.8	
Morbitdity (%)	63	69	58	62.5	36
POPF (%)	18.6	20.2	24	13.5	7.4
PPH (%)	20.2	NA	22	6.2	10
30d Mortality (%)	5.3	1.8	3	2.1	2.5
R0 Resection (%)	83.9	85	100	NA	95
Lymph node harvest	16.5	28	7	NA	16.3

Not surprisingly, most experienced centers show improved outcomes likely due to advanced learning curves. However, even within our early stage of the learning curve, we were able to cut down operating time, after having implemented optimized operating procedures

## Conclusion

The rising popularity in the health care setting as well as intense marketing of robotic-assisted techniques warrants its broader application and distribution, particularly expected in pancreatic surgery. While the implementation of novel techniques is indispensable for innovation in the surgical field, it sometimes involves unforeseeable risks. Important studies from centers that have already established large programs for robotic-assisted pancreatic surgery have indicated that good performance and major benefits are only achieved after a long learning curve [6, 7]. At this point, professional training centers have been implemented to train international surgeons in conducting safe robotic-assisted pancreatic procedures [13]. They facilitate the acquisition of proficient skills at the robotic console, prior to their application on patients. However, they are not able to provide immediate surgical guidance on step-by-step operating procedures, necessary to acquire consistent outcome parameters in complex surgical settings. In this manuscript, we emphasize and were able to show that institutional process optimization and standardization, may shorten such learning curves. For this reason, we strongly propose to broadly implement standard operating procedures, which may facilitate intra- and inter-institutional process optimization as well as providing guidance for novel centers to establish a robust, time efficient- and safe robotic-assisted pancreatic surgical program.

Considering the continuous development of this technology, such recommendations need to be regularly discussed and re-evaluated to establish national and international consensus.

## Data Availability

Not applicable.
